# Expression and activation of human endogenous retroviruses of the W family in blood cells and astrocytes: implications for the pathogenesis of multiple sclerosis

**DOI:** 10.1186/1742-4690-8-S2-P19

**Published:** 2011-10-03

**Authors:** Luciana Poddighe, Giuseppe Mameli, Alessandra Mei, Elena Uleri1, Caterina Serra, Roberto Manetti, Antonina Dolei

**Affiliations:** 1 Department of Biomedical Sciences and Center of Excellence for Biotechnology Development and Biodiversity Research; 2 Department of Clinical, Experimental and Oncological Medicine, University of Sassari, 07100 Sassari, Italy

## Background

Multiple sclerosis (MS) is a chronic inflammatory disease of the central nervous system, with demyelination and gliosis. Proposed pathogenic co-factors triggering MS pathogenesis are the Epstein Barr virus (EBV), and two elements of the W family of human endogenous retroviruses (HERV-W): MSRV, that forms free virions, and syncytin-1, the ERVWE1env protein; both retroelements have neuropathogenic properties. In the past we studied MSRV in MS patients in various temporal and clinical stages; in all cases, striking parallelisms between MS behaviour and MSRV/HERV-W presence/load were found. By simultaneous detection of MSRV and HHV-6, we found a direct correlation between MS and MSRV presence/load, but not for HHV-6. MS brains over-express MSRVenv and syncytin-1 transcripts, with respect to controls, while EBV presence was not detected.

## Materials and methods

Since late EBV seroconversion is a strong risk factor for MS development, we performed *in vitro* experiments on PBMC from MS patients and MSRV^+^ volunteers, as well as on U87-MG astroglioma cells, that were studied as such or were exposed to EBV or to recombinant EBV glycoprotein350 (EBVgp350), or to proinflammatory cytokines. The levels of MSRVenv and syncytin-1 mRNAs were evaluated by discriminatory real time RT-PCR assays. Flow cytometry was used to evaluate the HERV-Wenv protein on the plasmamembrane, as well the PBMC subsets.

## Results

Basal expression of MSRVenv and syncytin-1 occurs in astrocytes and in NK, B and monocyte cells, but not in T cells. This uneven expression is amplified in naive MS patients. Astrocyte infection by EBV and exposure to EBVgp350 stimulate the expression of HERV-W/MSRV/syncytin-1, with requirement of the NF-κB pathway. In EBVgp350-treated PBMC, MSRVenv and syncytin-1 are activated in B cells and monocytes, but not in T cells, nor in the highly expressing NK cells. The latter cells, but not the T cells, are activated by proinflammatory cytokines.

## Conclusions

The study demonstrates that there are interactions among the above proposed MS-cofactors. *In vivo*, a pathogenic outcome would depend on activation in abnormal situations/tissues, as it may occur in delayed EBV infection, or in the presence of particular host genetic backgrounds, or both.

